# Mr. Bailey's Case of Uterine Haemorrhage

**Published:** 1810-06

**Authors:** John Bailey

**Affiliations:** Harwich


### To Dr. SHEARMAN.

Dear Sir,

I Send you the accompanying obstetrical Case, to dispose of in the manner you think proper. Cases of this kind are at all times interesting to the practitioner, but particularly so when attended by occurrences out of the ordinary way.

Yours, &c.

JOHN BAILEY.

*Harwich, May* 12, 1810.

---

ON the 8th of March, about seven o'clock in the evening, a labouring man of the name of English, living in the parish of Little Oakley, four miles from my residence, waited upon me, to engage my attendance for his wife at her next lying-in; which, by his account, was to take place about the latter end of the month next ensuing. He called at that time of night, he said, because his wife was taken in an unusual way in the course of the day.

On questioning him, I learned that the woman, whilst engaged in her domestic avocations, had suffered slightly from uterine hæmorrhage; that she was attacked with no pain, and had no idea of her labour coming on. On that account my attendance was not then required, but I was to send her some medicine if it were deemed necessary, and the next day, if my business led me into her neighbourhood, I was to give her a call. The man took home with him, Acidi sulphur. gtt. x. Tinct. opii, gtt. xxx. T. digit. gtt. xij. Aq. carui. ℥iß. in a draught, to be taken at bed time, with this injunction, that if there should be a recurrence of the hæmorrhage to any extent, he was to lose no time in coming for me.

Hearing nothing from the patient, I did not see her till the next day. She informed me, that she had had no return of the discharge, but from the sensations which she felt, was apprehensive that it would return. I told her there was some reason to apprehend that it would do so, and that it was necessary, as she could not have immediate assistance in the event of its happening, to make use of every means towards its prevention. She was desired to keep herself as quiet as possible, to abstain from stimuli of every description, and to go to bed on the first appearance of the discharge. She promised to comply with these regulations.

From this day (March 9) I occasionally looked in as I passed the door, and found her busied about the concerns of her family, which consisted of five young children. On the 18th of the month she had a slight hæmorrhage. On the 22d, at five in the morning, her husband came to me, and requested my immediate attendance, as he was fearful, from the report of the women about her, that she would be dead before I could get to her. I went with as much expedition as possible, and found the woman in a very exhausted state. She was, however, capable of informing me that at four in the morning she was awakened with a pain that gave her some reason to believe labour was coming on; presently another pain followed, accompanied with a violent rushing discharge, which took away her senses: how long she continued thus, she does not know, but supposes that it did not exceed half an hour: on recovering, another discharge as copious nearly as the former came on, and then she sent her husband off for me.

She

She said she had not obeyed the injunctions the day before, which some time back she had received from me, but had done more for her family than she ought, and was certain that had she kept still and quiet, this would not have happened. She begged, if it were possible, she might have help to have it done instantly, as her life was going away from her. I told her if her situation was such as to admit of being relieved by delivery, that it should be done; but if not, that she must wait patiently, and keep herself still until the time should arrive, when she might be assisted. This being a fit opportunity, I made an examination into the state of the os uteri; it was high up, *not* dilated, *nor* dilatable; the cervix uteri was not obliterated; in this state of things, there was nothing to be done but wait patiently. As the poor woman had disobeyed the advice she before had received, I told her now, without reserve, that if she valued her life, and the future happiness of that family to which she was so desirous of administering present comfort, she would adhere to the opinions of those better able to judge than herself; and that if she persisted in doing as she had done, death would inevitably and suddenly ensue. The violence of this attack so alarmed her, that she promised to comply to the utmost with my desires: accordingly, she was enjoined to keep to the horizontal posture for the remainder of her pregnancy; to apply topical cold over the region of the uterus; and to abstain from food heated beyond a temperature of 50°; in short, to lay in a room without a fire, and to take drinks of the simplest kind, cold.

In the afternoon of this day, I rode over to see her again; no fresh discharge beyond an oozing had taken place; the following day there was no discharge at all, and she was free from pain of every kind. From this time to the third of April, nothing material occurred. Being in the parish on the evening of this day with another midwifery patient, and having some leisure, I thought I would step to see how she was; on my way thither, her husband, who had heard of my being near, met me, and told me that he was very glad to see me, as his wife was much worse. When I got to the poor woman, which was about ten at night, I found her extremely low and faint; her pulse was quick and weak, and her situation such as to excite a good deal of apprehension for her safety; in the course of the day she had had repeated small discharges unattended by pain.

I made an examination per vaginam, and distinguished the os uteri high up, a little dilated: the presenting part of the child I could not make out in the usual mode of examination, amination; and as the true state of every thing within reach in cases of this kind ought to be ascertained, I introduced my hand entirely. The os uteri was dilated to about four lines in diameter, and so ductile, that it admitted of extension to any degree; immediately over this, laid, not an edge or small part, but the whole body of the placenta.

Having satisfied myself of the true nature of the case, I withdrew my hand, and informed the woman that the time she so long had wished for was at length arrived, and that before morning, I hoped she would be safe in bed.—When she had collected about her all the female assistance which was thought necessary; after laying her in a proper situation, and cheering her with a little wine, I proceeded with caution in the arduous and painful task of risking life and reputation; the confidence, however, which an extensive practice in midwifery, and the repeated occurrence of many successful cases, gave me, led me on.

The os internum would have readily receded, had it not been confined by the placenta; for some time I sought to detach it, but it was expanded over so large a surface of the uterus, that I did not accomplish it. As I apprehended that a hæmorrhage from the vessels, arising immediately out of the uterus, would be sooner felt to the mother than if from the lesion of those seated in the body of the placenta; accordingly, I passed my hand through its substance, ruptured the membranes enveloping the child, carried my arm upwards, found the feet, and withdrew a living child. A very copious hæmorrhage accompanied the birth of the child, as is usual in cases of this description.

Thus far having gone on well, I felt no anxiety about future consequences, until the continuance of the discharge directed my attention to the secundines; in five minutes after the birth of the child, a most alarming discharge took place: the pulse sunk instantly; syncope came on. I placed my hand upon the abdomen, with the view of pressing and stimulating the uterus to contraction, and was surprized to find it not at all reduced in bulk: it was a twin case, and the unfortunate mother apparently in the very arms of death. There was no time for deliberation; the same practice to be pursued under infinitely more distressing circumstances as at first; another such loss would have sunk her beyond the power of human redemption. A second time I passed my hand through the lacerated substance of the placenta, and with the greatest difficulty ruptured the membranes of the second child; the feet were laid hold of and drawn down, and another living child soon extracted, extracted. The next objects to be obtained, were to bring away the placenta, and to excite the uterus to contraction; the first of these objects I effected by the third introduction of the hand, and the latter by the application of topical cold and pressure. All hæmorrhage, from the moment of the extraction of the placenta, ceased. The latter part of this operation was performed whilst the mother was in a state of exhaustion, as nearly approaching to death as possible.

Having completed all that was necessary to be done as to delivery, my attention was next directed to the mother; over the surface of the abdomen, cloths wetted with cold vinegar and water were applied; to the epigastrium warmth; and as soon as it could be procured, I got down by spoonfuls a small tea-cup full of red port, previously warmed. Persevering in this manner for about half an hour, I had the indescribable pleasure of feeling a pulsation in the artery at the wrist; in half an hour more it beat distinctly; respiration became regular, and in a few minutes she faintly articulated, "I am alive, thank God." *

Thus began and ended one of the worst flooding cases that ever came within the sphere of my practice. I do not know that much practical good is to be deduced from its history, the rules for practice in cases of uterine hæmorrhage being now so well understood, but if you be of opinion that it is worthy of record, it is very much at your service.

* The mother and children are at this time in perfect health.

